# The impact of wildfires on the diet of *Podarcis lusitanicus* revealed by DNA metabarcoding

**DOI:** 10.1371/journal.pone.0319238

**Published:** 2025-10-01

**Authors:** Catarina Simões, Diana S. Vasconcelos, Raquel Xavier, Xavier Santos, Catarina Rato, D. James Harris

**Affiliations:** 1 Department of Biology, Faculty of Science of the University of Porto, Porto, Portugal; 2 CIBIO – Research Centre in Biodiversity and Genetic Resources, Universidade do Porto, Campus de Vairão, Vila do Conde, Portugal; 3 BIOPOLIS; Program in Genomics, Biodiversity and Land Planning, CIBIO, Vairão, Portugal; Charles University: Univerzita Karlova, CZECHIA

## Abstract

Fire has long been recognized as an important ecological and evolutionary force in plant communities, but its influence on vertebrate community ecology, particularly regarding predator-prey interactions, remains understudied. This study reveals the impact of wildfires on the diet of *Podarcis lusitanicus*, a lizard species inhabiting a fire-prone region in the Iberian Peninsula. In order to explore diet variability associated with different local burn histories, we evaluated *P. lusitanicus* diet across three types of sites in Northern Portugal: those had not burned since 2016, those burned in 2016, and those more recently burned in 2022. *Podarcis lusitanicus* is a generalist arthropod predator with dietary flexibility. Given the turnover of arthropod species after fire, it is expected to find variations in diet caused by different fire histories, especially between unburned and recently burned sites. From DNA metabarcoding of faecal samples, our study revealed that while prey richness remained unaffected by wildfire regime, significant shifts occurred in diet composition between more recently burned and unburned areas. Specifically, we found that differences in diet composition between these two fire regimes were due to the presence of *Tapinoma* ants and jumping spiders (*Salticus scenicus*). These prey were present in the diets of lizards occupying unburned areas, while these were absent in areas burned in 2022. Interestingly, diets in unburned areas and areas burned in 2016 showed no significant differences, highlighting the lizards’ ecological flexibility and the habitat’s resilience over time. The ant species *T. topitotum* was found in dominance in both burned areas, suggesting that this species may be fire tolerant. In addition, families such as Cicadellidae and Noctuidae were found to be more associated with more recently burned areas. The use of DNA metabarcoding in this study was essential to provide a more detailed and accurate view of predator-prey interactions in ecosystems susceptible to fire, and therefore a better understanding of changes in prey consumption in this fire-adapted ecosystem.

## Introduction

Wildfires are a significant ecological disturbance, shaping the structure and dynamics of ecosystems worldwide [1]. They impact approximately one third of the Earth’s land surface [2], driving changes in species composition, habitat availability, and ecosystem functions [3,4]. Many ecosystems, including the Mediterranean vegetation, that is the subject of this study, have evolved traits to persist under specific fire regimes [5,6].Rapid changes in fire frequency and intensity pose critical challenges to species’ survival. Human activities, particularly those that increase fire recurrence, exacerbate these challenges, altering habitat conditions and threatening biodiversity [5,7]. Moreover, wildfires can have a direct effect, such as animal migration and/or mortality, and indirect effects on individual organisms by changing resource availability (e.g. food and shelter) and habitat structure with fires also often leading to the creation of a mosaic of fragmented habitats [8,9]. These changes in environment determine the temporal and spatial dynamics of the fauna and flora, both in the short and long term [9].

After a fire, the responses of faunal populations are likely to vary among species depending on the life history characteristics and time elapsed since the fire [8]. Wildfires rarely result in total mortality among vertebrates [10], especially for species adapted to cliffs and rocks, whose habitats typically remain intact after the fire [11,12], or for those with effective dispersal or burrowing abilities [13]. However, post-fire conditions can represent an increased risk due to reduced shelter and resources, and greater exposure to predators [14,15].

Fire has shaped predator-prey relationships for thousands of years [16], but with the increasing human-induced changes to Earth’s ecosystems, the interaction between fire and these dynamics now often leads to unpredictable and potentially harmful consequences [17]. In that sense, diet studies using DNA metabarcoding of prey can be useful to understand changes in trophic interactions mediated by fire occurrence, since they allow rapid and accurate identification of the taxa consumed by a predator. This is especially relevant in the current scenario of increased wildfires, to understand the impact of these alterations on the prey communities and how these affect the predator populations [18].

Portugal periodically faces serious damages due to wildfires [19,20]. Climatic conditions, such as episodes of high rainfall followed by dry periods, coupled with strong winds, significantly increase the likelihood of natural wildfires in this country [21–23], especially in Northern Portugal where most of the country’s burned areas are found [24]. However, in recent decades, socio-economic changes have shifted the primary causes of fire ignition from natural to predominantly anthropogenic sources. Human activities, including deliberate actions, negligence, accidents, and carelessness, have been responsible for 97% of wildfire ignitions in Portugal over the last three decades [22]. Additionally, the composition of Portuguese forests increases the risk of fire, as they are largely dominated by highly flammable monocultures of pine (*Pinus pinaster)* and eucalyptus (*Eucalyptus* spp.), whose essential oils make them particularly prone to burning, whereas native oak species (*Quercus* spp.) are better adapted to fire regimes and less flammable [25].

Lacertid wall lizards of the genus *Podarcis* (Wagler 1830), are a group of reptiles that evolved and diversified along the Mediterranean Basin [26,27]. They represent a principal herpetofauna element of Mediterranean ecosystems, playing an important ecological role in food chains [28]. The Lusitanian wall lizard *Podarcis lusitanicus* is endemic to the Iberian Peninsula, being widespread in northern Portugal, northwest Spain, and northeast to Picos de Europa [29], and whose demographic history was highly influenced by the Pleistocene glaciations [30]. *Podarcis lusitanicus* is a saxicolous, diurnal and small lizard that tends to use walls, logs and areas with less vegetation for thermoregulation, foraging and shelter [26]. This species is insectivorous, feeding on a wide variety of terrestrial invertebrates. Across its Portuguese range, this lizard has an irregular distribution, with populations located on open natural rocky outcrops and artificial stone walls surrounding agricultural fields [31]. Because of its preference for open rocky areas [32], *P. lusitanicus* populations can be important in landscapes which experience recurring wildfires. [33,34]. Fire removes the vegetation and reveals rocky outcrops, creating new habitats, which favours population expansion, dispersal between metapopulations and the recruitment of migrants, increasing the population genetic diversity [21]. The high rainfall rate in northern Portugal leads to a fast vegetation recovery that is prone to become burned again [35]. For lizards living in areas with recurrent fires, fire-vegetation-recovery cycles cause prey availability to change, depending on the time since the last fire. However, while some taxa show resilience to fire disturbance, others, including prey species of predators tolerant to fire like the wall lizard *P. lusitanicus*, can be highly sensitive to fire [16].

In this study, we assessed the impact of fires on the diet composition and diversity of the Lusitanian wall lizard based on a DNA metabarcoding approach. We described the diet of lizard populations living in recently burned plots (one year since the last fire), plots burned seven years since the last fire, and long-unburned plots. Given the high species turnover among arthropod groups after fire [36–38], we expect significant differences in the composition of lizards’ diets, especially between plots of the most different fire history.

DNA metabarcoding was chosen for this study because it enables the identification of nearly all prey species consumed, including rare or soft-bodied organisms that may not leave identifiable remains for traditional visual analysis [39–41]. Unlike morphological methods, which depend on intact specimens and specialized taxonomists, metabarcoding can accurately identify prey even when they are damaged, poorly preserved, or in immature stages [42]. While it does not provide information on the relative abundance of prey, it ensures a comprehensive and thorough dietary assessment based on the available DNA, addressing key limitations of conventional identification techniques.

## Materials and methods

### Study area

**Fig 1:**
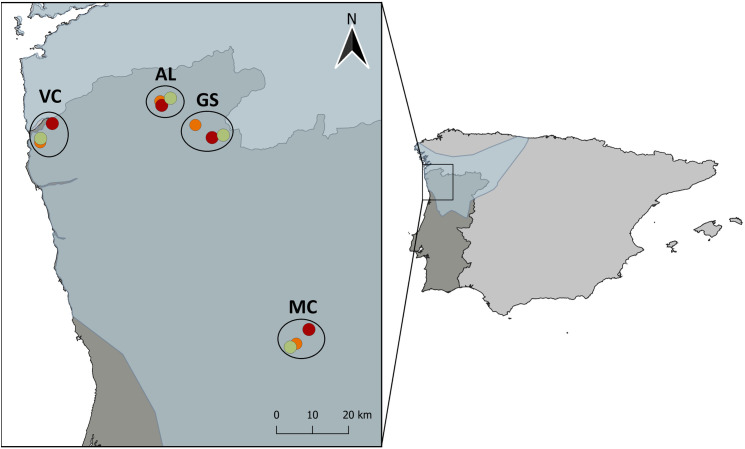
Location of the study sites in the NW extreme of Portugal. Each sampled location - Viana do Castelo (VC), Álvora (AL), Gerês-Soajo (GS) and Marco de Canaveses (MC) – is comprised by three sites with different historic fire conditions: unburned (UN in green), burned in 2016 (BU16 in orange), and burned in 2022 (BU22 in red). All sites with different fire conditions were at least 1400m apart, ensuring no lizards foraged between them. The approximate distribution range of *P. lusitanicus* is shown in blue, on the right map. Distribution of species *P. lusitanicus* republished from IUCN (2025) [43] under a CC BY license, with permission from International Union for Conservation of Nature, original copyright 2025.

This study was performed in northwestern Portugal confined by the latitudes 41º58’ to 41º09’ N and longitudes −8º49’ to −7º56’ W (Fig 1).

Four locations, Viana do Castelo (VC), Marco de Canaveses (MC), Gerês-Soajo (GS) and Álvora (AL), were chosen based on habitat suitability and similarity (e.g. topography) and fire occurrence (ICNF; https://geocatalogo.icnf.pt/catalogo_tema5.html). At each location we selected an area burned in 2022 (coded as BU22), another burned in 2016 (coded as BU16), and a long-unburned area (coded as UN; no fires recorded since 2016 onwards), totalling 12 sampling sites (Fig 1), that include four replicates for each of the three fire regime groups (BU16, BU22 and UN). The selected sites, BU16, BU22, and UN, were located within at least ~1.4 km and up to ~3.2 km of each other, ensuring independent foraging areas at each location, since home ranges are typically less than 500 m² [33], implying range diameters of less than 30m, and exhibited similar overall landscapes, including rocky habitats where these lizards inhabit. To evaluate differences in vegetation structure across fire regime sites, we used the Normalized Difference Vegetation Index (NDVI), a metric commonly used to estimate primary productivity and green biomass [44], and particularly suitable for detecting fire-induced landscape changes [45]. NDVI values were derived from cloud-free Landsat satellite images (30 × 30 m resolution) downloaded from the U.S. Geological Survey (https://www.usgs.gov/), covering the period from May 1st to June 30th, 2023, which coincided with lizard field surveys. We extracted NDVI values within 100 m radius buffers around each sampling site and, at the plot level, measured the values of nine pixels (3 × 3 window) to capture local variation. A Generalized Linear Mixed Model (GLMM) with Gaussian error distribution was used to test whether NDVI values differed among fire regime sites, with fire regime site as a fixed factor and locality as a random factor, using the RRPP R package [46].

### Study species

Members of the Lacertidae family are typical examples of generalist species, feeding on a high variety of terrestrial invertebrates [47,48]. Previous studies on the diet of *P. lusitanicus* have used morphological prey identification through stomach contents [49,50] or pellets [51]. They revealed that the main prey groups included Hemiptera, Coleoptera, Araneae, Hymenoptera and Diptera. To a lesser extent, Orthoptera, Lepidoptera (including larvae), Opiliones, Miriapoda, Isopoda, Collembola, Diptera larvae, Trichoptera larvae, Mecoptera, Blattodea, Thysanoptera, Dermaptera, Embioptera, Oligochaetes and Gastropods were also identified, as well as vertebrates [49,50,52–54].

In relation to wildfires, while certain taxa are resistant to the disturbances caused by fire, others are highly susceptible to its effects [16]. Arthropods, particularly in their immobile larval stages, are generally vulnerable, with poor dispersers being at greater risk [36]. Many insects, due to their flight capacity, are able to quickly recolonize affected areas, while surviving arthropods may benefit from the post-disturbance environment through diminished competition or a reduced risk of predation [55,56]. Ants, for instance, demonstrate remarkable resilience by relocating to unburned areas, including underground refuges, which minimizes fire-related mortality [57]. In general, omnivorous fauna, including predators, tend to be more resilient and recolonize disturbed areas more quickly, as they are not reliant on specific trophic resources, allowing them to adapt to shifting food availability [58] and focus on the most abundant and easily captured prey species [17]. Grasshopper species may also temporarily increase in abundance in recently burned plots, as their generalist and invasive traits, combined with strong dispersal capabilities, allow them to thrive in post-fire conditions [57].

### Sampling methods

Sampling of *Podarcis lusitanicus* lizards was carried out during the day (10 AM – 3 PM) between May and June 2023, across natural open rocky outcrops and rock walls of abandoned agricultural fields.

Experimental protocols and research were approved by the Portuguese Institute for Conservation of Nature and Forests (ICNF) (License 552–553/2022/CAPT). No animal experimentation was performed in this study. Lizards were captured with a noose. Generally, lizards defecated soon after capture, otherwise a simple abdomen massage was enough to obtain a freshly voided faecal sample. Faecal pellets were collected from 183 individuals of *P. lusitanicus*: 38 in Viana do Castelo, 50 in Marco de Canaveses, 53 in Álvora and 42 in Gerês-Soajo (S1 Table). All faecal samples were preserved in 96% ethanol.

Sex was determined by the presence of developed femoral pores, and the robustness of the head [59–61]. We also took standard morphological measurements, including weight and snout ventral length (SVL) using a digital scale and calliper, respectively. All measurements were taken by the same person (D.S. Vasconcelos) to eliminate inter-observer error. No animals died or were euthanized during sampling and all animals were released unharmed at the capture site on the same day.

### DNA extraction and amplicon library preparation

The 183 pellet samples were extracted using the E.Z.N.A. Tissue DNA Kit (Omega Bio-Tek, U.S.A.), following the manufacturer’s instructions with a minor modification in the digestion step, using 800 μL of Gordon buffer instead of the 200 μL of TL buffer (from the kit) to improve DNA extraction of both hard and soft tissues [62]. All samples were vortexed to disrupt the faecal mass and digestion occurred over 3 hours. Extracted DNA was stored at −20ºC. Each extraction set included a single negative control, in which ultra-pure water was used for PCR instead of DNA extract, to control for contaminants present in extraction kits and/or in the laboratory environment, resulting in a total of eight extraction negative controls. A short fragment (~205 bp) of the mitochondrial cytochrome c oxidase subunit I (COI) was amplified using Polymerase Chain Reaction (PCR) with the Fwh2 primers (FwhF2 and FwhR2n) developed by Vamos et al. (2017) [63], which showed effective performance in metabarcoding terrestrial arthropod samples [63–65]. Primers were modified to include Illumina adaptors and a 0–5 bp addition of N bases between the adaptor and the primer to increase sequencing diversity and quality [66–68]. The different primer variations were then combined before PCR reactions, resulting in mixed forward and reverse primer single solutions.

The PCR reaction consisted of 5 μL of QUIAGEN Multiplex PCR Master Mix (Quiagen, Crawley, UK), 0.3 μL combination of six forward primers, 0.3 μL combination of reverse primers, 2.4 μL of ultra-pure water, and 2 μL of DNA extract. Cycling conditions used consisted of an initial denaturation at 95°C for 15 min, followed by 45 cycles of 95°C for 30s, annealing at 52°C for 45s, extension at 72°C for 20s, and a final extension at 60°C for 5 min. Four PCR negative controls were used to detect possible contaminations in the PCR reagents.

An initial PCR clean-up to remove unused primers and primer dimers was performed, using the Agencourt AMPure XP beads (Beckman Coulter, Brea, CA, USA). This cleaning was performed using a proportion of 0.85 μL of magnetic beads to 1 μL of PCR product. To attach unique barcodes and Illumina sequencing indexes (Illumina, San Diego, CA, USA) to each sample, a second PCR was performed using a distinct combination of index sequences per sample. This PCR index was performed using 2.8 μL of ultra-pure water, 7 μL of 2x Kapa HiFi, 1.4 μL of each index, and 2.8 μL of cleaned DNA. Indexing the PCR required an initial denaturation of 95°C for 3 min, followed by 10 cycles of 95°C for 30s, annealing at 55°C for 30s, extension at 72°C for 30s, and a final extension of 72°C for 5 min. In order to confirm the success of the barcode’s incorporation in all samples, PCR products were tested in a 2% agarose gel. A second PCR clean-up was then performed, under the same conditions as the first one, except that the beads’ ratio was 0.8.

After the second PCR clean-up, all indexed PCRs were quantified using Epoch™ Microplate Spectrophotometer (BioTek Instruments, Inc.; Winooski, VT, USA), followed by the normalisation to obtain the same concentration in all the samples. After the normalisation, the samples were pooled together. The pool was tested for quality control in a TapeStation 4200 High Sensitivity D1000 Assay (Agilent, Santa Clara, CA), and a cleaning was needed using a 0.78 ratio of beads. A second test for quality control in the TapeStation was performed to confirm the success of the cleaning. The final pool was sent to GENEWIZ Next Generation Sequencing laboratory with a final concentration of 27nM to be sequenced in an Illumina MiSeq sequencer with 2x250bp paired-end (PE) configuration and PhiX (≤30%) was used to increase sequencing diversity.

### Sequence denoising and taxonomic assignment

The software PEAR [69] was used to merge the forward and reverse reads into single sequences, discarding single and unassembled reads. Base pairs with less than 26 bp of overlap were also rejected. Then, the primers were removed, using the command *ngsfilter* from OBITools [70]. The reads were once more counted to check for successful rates of the previous step with the *grep* command and then dereplicated into unique sequences using the *obiuniq* command. Lastly, sequence cleaning was performed to remove sequences with less than 10 reads and chimeras, using the *obiclean* command. Sequences with a length between 202 bp and 208 bp were kept and clustered at a 99% similarity threshold to form Operational Taxonomic Units (OTUs), using VSearch [71]. OTUs that were represented by a read count < 1% of the total number of reads obtained for each individual sample were removed [72]. This should allow the removal of most PCR and sequencing errors that still passed the *obiclean* denoising step. Additionally, all reads identified in the extraction and PCR controls were subtracted from the corresponding sample batch [73].

The obtained OTU table and sequences were further cleaned to remove redundant and unreliable OTUs using the R package LULU [74], and the taxonomic assignment of OTUs was performed using BOLDigger [75], followed by manual inspection and curation. We also used the BLAST function from NCBI implemented in Geneious Prime 2021.1.1 [76], when species-level assignments were not possible with BOLDigger. The taxonomic level assignment was performed in both search engines according to the following thresholds: to the class level if below 90%; to the family level if between 90 and 95%; and to the species or genus level (in cases where more than one species from the same genus had a > 95% sequence match) if higher than 95% similarity. In the BLAST results, similarity corresponds to the “percent identity”, while in BOLDigger this metric is called “ID%”. Sequences identified as non-target amplifications (S2 Table) were excluded from downstream analyses. These included host DNA (*Podarcis lusitanicus*), environmental contaminants (e.g., fungi), and sequences matching symbiotic or parasitic organisms (e.g., nematodes).

Following the completion of taxonomic assignments, the distribution and presence of detected prey species in Portugal were verified using a combination of local databases, that included Naturdata (https://naturdata.com/) and Lusoborboletas (https://www.lusoborboletaspt.com/) and global databases, such as GBIF (http://www.gbif.org/). Additionally, relevant scientific articles were reviewed to confirm whether the presence of specific species within Portuguese ecosystems had already been documented.

### Statistical analyses

Lizards body size (snout-vent length, hereafter SVL) and weight (hereafter BW) can influence diet composition [51,77]. For this reason, we compared SVL and BW between sexes and among fire sites. First, the normality of BW and SVL distributions was assessed using the Shapiro-Wilk test, which revealed that while SVL (p = 0.452) followed a normal distribution, BW did not (p = 0.0199). Consequently, both SVL and BW were log-transformed. Variation in logSVL and logWeight across sexes and fire sites (burned in 2016, burned in 2022, and unburned) was visualized using boxplots. Body condition was analysed through a linear regression between logWeight and logSVL for males and females. An analysis of variance (ANOVA) was conducted using the *aov* function from the stats package v. 3.6.2 [78] to examine sexual dimorphism in body size (logSVL) while accounting for Fire site and Sex as fixed factors, and locality as a random factor. Similarly, a separate ANOVA was conducted to analyse the variation in body mass (logWeight) using the same fixed and random factors. However, since logWeight and logSVL are correlated, subsequent analyses focused only on logSVL. Pairwise comparisons were carried out using the emmeans package 1.10.2 [79].

For the statistical analysis, R 4.1.0 [78] was used to assess differences in dietary descriptors (i.e., diet richness and composition). Dietary analysis was based on two different taxonomic levels: OTU (all taxonomic units identified to the lowest resolved taxonomic level) and family. For the OTUs not identified to the species level, we built a neighbour-joining tree in Geneious Prime v. 2024.0.2 (Biomatters), visually inspected the corresponding alignment, and checked for patterns of genetic similarity (~98%) in order to cluster them into distinct taxa (e.g., Carabidae 1, Carabidae 2, and so on).

A Generalized Linear Mixed Model (GLMM) was implemented to investigate the effects of different predictors on diet richness (i.e. the number of different OTUs or family prey types per sample) while accounting for the replicate structure of the sampling scheme. This approach was chosen because the number of OTU or family prey items per pellet followed a Poisson distribution, as determined by the Shapiro-Wilk test. Fire site, sex, and logSVL were included as fixed effects, while Locality was considered as a random factor. Pairwise comparisons were again carried out using the emmeans package. The magnitude (effect size) and statistical significance (positive or negative) of the predictor variables on OTU richness and family richness were analysed by presenting the model coefficients and their respective 95% confidence intervals. When the confidence intervals include the value zero, this indicates that the effect is not statistically significant (p ≥ 0.05).

The iNEXT v. 3.0.1 package [80] was used to perform rarefaction and extrapolation curves to analyse whether the type of fire (unburned versus burned; burned in 2016 versus burned in 2022) and Sex had any impact on both the OTU and family richness, setting the confidence level at 84% [81] with 1,000 bootstrap replicates. Further, using the *ggiNEXT* function both sample-based and coverage-based rarefaction curves were plotted. However, the estimated richness was compared considering completeness (i.e., sample coverage) instead of sample size (i.e., number of samples), to avoid biases of communities with different levels of richness requiring different sampling efforts to be sufficiently characterised [82]. This is especially important for diet analysis in this generalist species, as its diverse diet can lead to varying richness levels.

Additionally, we used rank abundance to compare the top 10 most abundant prey species across the different Fire sites, using the *ggplot* from the ggplot2 package in R [83]. However, it is important to note that these frequencies reflect the occurrence of prey species in the samples rather than their actual abundance, as metabarcoding provides only presence/absence data and does not quantify the actual abundance of each prey type in the diet.

To compare diet composition at the OTU and family levels, a permutational multivariate analysis of variance (PERMANOVA) was used with Fire site, Sex and logSVL as fixed factors, using the R package vegan 2.6–6.1 [84], considering a stratified design (strata = Locality). The presence or absence of each prey item in each sample was used to build a Jaccard (among OTUs) and Bray-Curtis (among Families) similarity index using the *vegdist* function from the vegan package. After the PERMANOVA, a pairwise comparison was performed using the *pairwise.adonis2* function from the pairwiseAdonis R package [85].

A homogeneity of dispersion test (function *betadisper*) was performed to make sure that the differences observed with PERMANOVA were not due to unequally dispersed values across the different groups, as opposed to differences in group centroids, automatically adjusting the p-values. Afterwards, a similarity percentage analysis was carried out, using the function *SIMPER* implemented in the vegan R package, to assess the contribution of each prey item to the differentiation between diets.

Finally, a redundancy analysis (RDA) was done using prey types (Families) that occurred in over 5 samples (i.e., were found in at least 5 different individuals) using sex and Fire site as factors and locality as conditional to account for spatial heterogeneity. This analysis allows us to identify associations between specific prey Families with a particular fire site. The RDA was performed using the vegan package, and scaling was applied to standardize the data. A separate permutation test was run for each explanatory term to evaluate their individual contributions to the model.

## Results

The GLMM revealed a significant effect of fire regime on NDVI values (p < 0.001), indicating that unburned sites consistently exhibited higher NDVI values compared to burned sites, across both 2016 and 2022 (S3 Table). Locality also showed a significant effect (F = 11.406, p < 0.001), suggesting that NDVI varied across different sites. However, since locality was treated as a random factor, this variation was accounted for in the model structure.

The regression for logSVL and logweight shows that these parameters are significantly and positively correlated in both females and males (Females: r2 = 0.427; Males: r2 = 0.491), with males being larger and heavier than females (S2 Fig). The only parameter that significantly affects SVL (p = 0.0116) is Sex, but the logWeight is significantly influenced by both Sex (p = 3.72e-05) and Fire site (p = 0.0201) (S3 Fig and S4 Table). The significant differences in weight obtained between the Fire regime sites were due to differences between recently burned areas (BU22) and unburned ones (UN) (p = 0.0251), with lizards from recently burned sites exhibiting significantly higher body weights than those from unburned sites (S5 Table).

### Sequence filtering

Approximately 23 million raw sequence reads were generated from the 183 faecal samples analysed. 357,866 reads were retained after quality control, resulting in 1,102 OTUs. Of those, 80 OTUs, representing 9.4% of total reads, did not match any reference OTUs in the database used. Non-target amplification (37.39% of total reads) was observed from different sources, both in the samples and in the extraction and PCR negative controls. Most of the non-target OTUs were identified as belonging to Fungi (mainly Ascomycota and Basidiomycota), accounting for 12.53% of total reads and 33.51% of non-target OTU diversity. An expected presence of *Podarcis lusitanicus* (6.88% of total reads) was observed. The final dietary dataset comprised 237,402 reads, encompassing 537 distinct OTUs.

### Diet analyses

From a total of 537 prey items, five classes of Arthropoda (Arachnida, Collembola, Diplopoda, Insecta and Malacostraca), one class of Mollusca (Gastropoda), and one class of Annelida (Clitellata), were identified. These encompassed at least 26 distinct orders, 95 families and 93 species (S6 Table), with the majority of unique OTUs belonging to Arthropoda. In general, the order Araneae was the most frequently detected prey, occurring in 21% of the samples, with Salticidae being the most frequent family (10%). The other most frequently consumed prey belonged to Coleoptera (12%), Hemiptera (12%), Hymenoptera (11%) and Lepidoptera (7%) (S4 Fig; S7 Table).

The GLMM showed that none of the variables Fire regime site, Sex and SVL (logSVL) had a significant effect on either OTU or family diet richness (S8 Table). The estimated model coefficients and 95% confidence intervals for OTU richness (S5A Fig) and family richness (S5B Fig), indicate that the confidence interval for Fire regime site, Sex and LogSVL overlap zero, representing no significant effects among groups.

**Fig 2:**
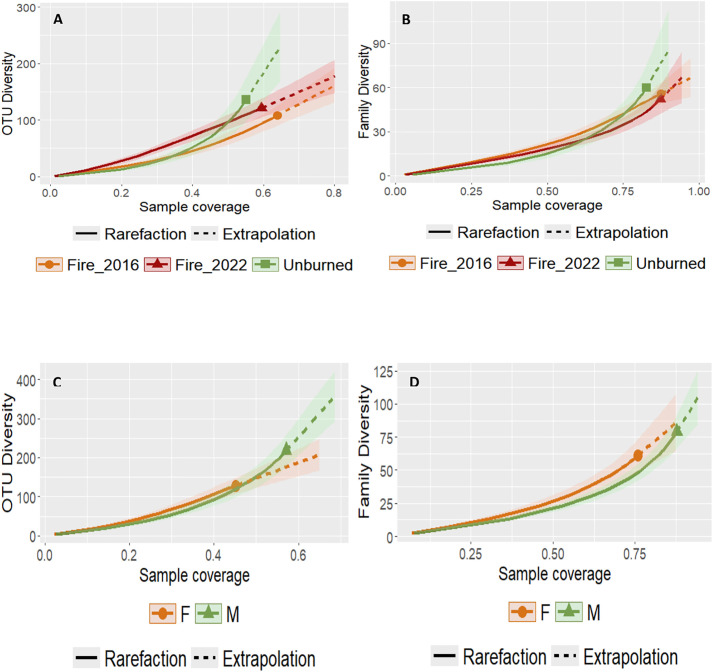
Diet rarefaction curves for (A and B) fire regime site (Bu16, BU22 and UN), and (C and D) sex (F, female; M, male), showing the observed (full line) and estimated (dashed line) OTU (A and C) and family (B and D) richness, and their respective 84% confidence intervals by sample coverage.

From the analysis of the rarefaction and extrapolation curves (Fig 2), we observed no difference between regime fire sites, at both the OTU and family levels (Fig 2A and 2B). The same pattern is observed between sexes (Fig 2C and 2D). For all rarefaction analysis, the minimum observed sample coverage was always above 50%, except for the unburned areas in the OTU diversity analysis (Fig 2A) and the Females (F) in the OTU diversity analysis (Fig 2C).

The rank abundance plot (S6 Fig) shows that the most abundant species present in both burned areas is the terrestrial amphipod *Talitroides topitotum*, while in the unburned areas the most abundant OTU was an unidentified taxa of Arachnids (“Arachnida 2”), followed by *T. topitotum*. The BU22 line shows a steep drop after the first most abundant OTU, indicating high dominance by a few species.

The PERMANOVA results indicated that Fire regime site significantly influenced the OTU community composition (p = 0.014), while Sex and SVL did not have significant effects (Table 1). The homogeneity of dispersion test confirms that this is not due to unequally dispersed values (F = 0.8968, p = 0.4097). The significant differences obtained between the Fire regime sites were due to differences between recently burned (BU22) and unburned areas (UN) (p = 0.004; Table 1), although the differences between the unburned areas (UN) and the areas burned in 2016 (BU16) were nearly significant (p = 0.051; Table 1). The OTUs *Salticus scenicus* and “*Tapinoma* sp.1” contributed the most to the differences found in PERMANOVA between the UN and BU22. At the family level, the PERMANOVA did not show significant effects of any of the considered factors in diet composition (Table 1).

**Table 1:** PERMANOVA results for OTU and family community composition and Pairwise PERMANOVA Comparisons for OTU by Fire regime site. Significant p-values (p < 0.05) are highlighted in bold.

	Factor	Df	Sum of Squares	R²	F	p-value
	Fire regime site	2	1.400	0.01622	1.4672	**0.014**
**OTU**	Sex	1	0.366	0.00424	0.7676	0.872
	logSVL	1	0.563	0.00652	1.1792	0.437
	Fire regime site	2	1.340	0.01714	1.5125	0.083
**Family**	Sex	1	0.433	0.00554	0.9780	0.400
	logSVL	1	0.664	0.00850	1.4995	0.244
	BU 16 vs BU 22	1	0.593	0.01075	1.2389	0.116
**OTU by Fire regime site**	BU 16 vs UN	1	0.675	0.01203	1.4248	0.051
	BU 22 vs UN	1	0.821	0.01354	1.7162	**0.004**

**Fig 3:**
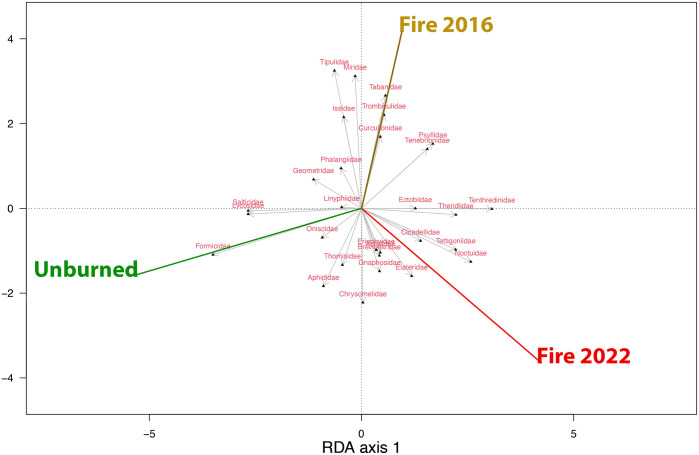
Redundancy analysis (RDA) biplot showing the relationship between prey abundance per family and fire history. Arrows represent arthropod families, with their orientation and length indicating their contribution to the variation explained by the first two RDA axes (RDA1 and RDA2). Only arthropod families met the threshold of occurring in at least five samples and were therefore included in the analysis. Coloured vectors represent plot types: unburned (green), burned in 2016 (yellow) and burned in 2022 (red).

According to the RDA, fire regime site had a statistically significant effect on family abundance (p = 0.045; S9 Table), while sex did (p = 0.546; S9 Table). From the results it was possible to observe specific associations between prey families and fire regime sites: Miridae, Tabanidae and Curculionidae were associated with areas burned in 2016; Cicadellidae and Noctuidae were associated with areas burned in 2022; and Formicidae and Salticidae were associated with unburnt areas (Fig 3). No single RDA axis was individually significant, although the first two axes captured the majority of the constrained variance (40.2% and 38.6% for the first and second axis, respectively).

## Discussion

This study assessed the effect of wildfires on the trophic niche of *Podarcis lusitanicus* by comparing diet richness and composition among specimens captured at recently burned (in 2016 and 2022) and unburned sites. Diet composition varied across sites, with significant differences in lizard diet composition between the most recently burned (2022) and unburned sites. These differences were predominantly associated with differences in prevalence of certain Arthropod taxa. Diet richness did not change significantly between sites or according to sex or body length.

Our results confirmed that the diet of *P. lusitanicus* is quite diverse, with a taxonomic composition in line with previous morphological studies carried out on this species, highlighting the predominant groups, such as Hemiptera, Coleoptera, Araneae, Hymenoptera and Diptera [86]. However, a far greater diversity of prey items was identified in our study compared to previous ones [50,86]. This study revealed 8 additional orders not previously identified: Trombidoformes, Sarcoptiformes and Mesostigmata (Arachnida); Psocodea, Plecoptera, Odonata and Archaeognatha (Insecta); and Amphipoda (Malacostraca), with the latter corresponding to all OTUs identified as *Talitroides topitotum*. These orders include small species that could easily go unnoticed in morphological analyses. The orders Trombidiformes, Sarcoptiformes, Mesostigmata and Psocodea include parasitic species, particularly among mites and lice. Therefore, the presence of these orders may be a result of secondary consumption, i.e., through host consumption, not necessarily reflecting the diet of the lizards. Of these orders, only Odonata contains larger species that, in principle, would not go unnoticed in morphological analyses. However, as it was found in only two samples in this study, it can be considered a rare occurrence.

Contrary to the genetic approach, diet studies based on morphology tend to underestimate the presence of soft-bodied prey, as they typically only detect items that have not been completely digested [87]. The significant increase in the number of orders identified in this study (26) compared to previous studies using microscopy (15 and 19 in Pérez Mellado (1982) [50] and Carretero et al. (2022) [86], respectively) suggests that the prey spectrum of *P. lusitanicus* is considerably larger than initially reported. In addition, this metabarcoding study provided greater precision in the taxonomic identification of prey, allowing a great number of them to be identified up to the species level (93 see S10 Table), which is usually not possible in studies based on morphological scatology.

### Fire impact on richness and composition

Our results demonstrated that lizards in burned areas seem to be able to maintain levels of dietary diversity. This can be explained by the ability of these lizards to feed on invertebrate species that may be more resilient to fire or those that quickly colonise habitats after the fire. Lacertid lizards are known to show remarkable dietary flexibility in response to environmental changes. Although their diet composition is not determined solely by prey availability [28], they can change their diet based on the type of habitat and resources available after a disturbance [48].

Wildfires in Mediterranean areas cause the fragmentation and destruction of original forest habitats, resulting in a decrease in the size of these areas, increasing the isolation of forest patches and creating a heterogeneous landscape. This creates forest archipelagos dispersed in matrices of open or shrubby vegetation, with different post-wildfire ages [9]. In these open landscapes both vegetation and fauna recover through ecological succession, from low xeric shrubs to tall evergreen forests [88–90]. As a consequence, differences in prey items between areas affected and unaffected by fire would be expected [91]. Essentially, the recovery of prey communities, such as arthropods, after major disturbances like wildfires, can be attributed to two main factors: their resistance to fire, which is their ability to survive when flames pass through, and their resilience, which includes their capacity to recolonize burned areas, take advantage of new resources created by the fire, and adapt as the habitat gradually recovers to its original state [38,92,93]. Refugia not only increases immediate survival during a fire but can also facilitate the persistence and recovery of populations in affected areas. In the long term, as vegetation regenerates, these refugia can help re-establish populations, acting as sources of expansion within the burned area or facilitating the arrival of new individuals from outside that area, providing both short-term (food and shelter) and long-term (permanent habitat) resources [92,94,95]. In this study, we demonstrated that depending on the fire regime, diet composition is significantly different, corroborating the previously described trophic behaviour for a generalist species like *P. lusitanicus*. Specifically, the differences are observed between areas recently burned in 2022 and unburned areas, i.e. those fire regimes supporting the highest structural differences in the habitat, as confirmed by NDVI-based GLMM results. These differences in diet composition were mainly due to the presence of the ant *Tapinoma* sp.1 and the spider *Salticus scenicus* in unburned areas, and their total absence in the diet of the lizards from the 2022 burned sites. The results of the RDA suggest that other prey types also contribute to dietary differences among lizards from different fire regime sites, suggesting a more complex pattern of prey selection. The absence of significant differences in diet composition at the family level suggests that, although fires can alter the composition of specific OTUs, the broader taxonomic structure (e.g., consumption of ants, spiders, etc.) remains relatively stable.

The finding that neither diet richness nor composition differ significantly between males and females suggests that both sexes have similar access to food resources and use them in a similar way, possibly due to a shared ecological niche or similar behaviours. A previous study in *Podarcis* has demonstrated that prey size selection, but not richness, can be influenced by morphological characteristics with males selecting larger prey items, a tendency that correlates positively with head size and bite force, both bigger in males than in females [51].Considering that the studied populations of *P. lusitanicus* show sexual dimorphism for both size and weight, a more comprehensive overview of the diet provided by a metabarcoding approach, could have revealed previously unnoticed differences in prey composition between sexes. However, our dietary assessment confirms the absence of sexual differences in prey richness. Since faecal samples were crushed during DNA extraction, we are unable to verify whether, despite consuming similar taxonomical groups of arthropods, males and females of *P. lusitanicus* are selecting specimens of different sizes or even from distinct ontogenic stages.

### Fire responsive taxa

Wildfires can significantly impact fire-sensitive species, like some of the prey items in this study, causing population declines immediately after a fire (e.g., [11]). However, complete local extinctions are rare, as survival depends on fire intensity, the availability of refuges, and species-specific avoidance behaviours [94,96,97]. Habitat conditions can also be altered by fire, with a reduction in soil moisture and organic matter, which are essential for many arthropods [98]. Changes in temperature and resource availability can disrupt life cycles, causing species to migrate or adapt to new conditions [99–101]. As a result, community composition shifts, with early post-fire succession favouring open-habitat species that can disperse over long distances, while unburned areas continue to support species dependent on dense vegetation [102–104]. Therefore, the effect of fire on species richness is highly variable and depends on habitat type, fire intensity, and the taxonomic group considered. For example, different ant taxa can be classified as “fire-tolerant”, “fire-intolerant” or “fire-neutral” depending on their ability to survive and adapt to fire-disturbed habitats. Interestingly, previous studies, such as the one from EL Khayati [57], reported changes in ant species between burned and unburned areas, matching with the findings of our study where lizards in unburned plots consumed a higher diversity of Formicidae. Similarly, Salticidae (jumping spiders) were also predominantly foraged by lizards in unburned habitats. Despite this association, spiders and ants are often considered moderately fire-resistant due to traits that enhance their survival, such as burrowing behaviours, the ability to utilize refuges like logs and rock piles, and their capacity to adapt to post-fire environments [98,105]. However, the association of these taxa with unburned areas as observed in this study, does not necessarily indicate a decrease in their abundance in burned areas, since this research did not examine pre- and post-fire arthropod diversity directly.

Families such as Cicadellidae (Hemiptera) and Tettigoniidae (Orthoptera) are known to be frequently more abundant in burned areas, where early successional regrowth provides suitable conditions, such as fresh leaves for Cicadellidae and open spaces with new vegetations for Tettigoniidae [106,107]. Not surprisingly, our results showed both Cicadellidae and Tettigoniidae were abundant prey items in the diet of lizards from burned areas. Similarly, in plots burned in 2016, the families Tabanidae, Miridae, and Curculionidae were also more abundant in lizards diet. Tabanidae, as pollinators, and Miridae and Curculionidae, as plant feeders, likely benefit from the increased availability of nectar and plant material in regenerating landscapes [106,108]. Notably, Miridae has been reported to maintain high abundances in both burned and unburned sites, likely due to their ecological flexibility to a wide range of vegetation [106,109].

The dominance of *Talitroides topitotum* in both burned areas suggests that this species may be a fire tolerant species. *Talitroides topitotum* is a terrestrial amphipod native to Asia, widely found in subtropical and temperate regions around the world [110]. The species has been introduced to countries such as Costa Rica [110,111], Brazil [112] and Indonesia [113] and is able to adapt to diverse temperature and humidity conditions [110]. Due to its high capacity for dispersal and establishment in new environments, *T. topitotum* can become potentially invasive in various habitats, posing a threat to native amphipod species [110,113]. *Talitroides topitotum* also seems to be associated with areas that were reforested with *Eucalyptus* spp., a culture that covers extensive areas in Brazil [114] and that represents the most widespread tree species in northern Portuguese mainland [115]. To the best of our knowledge, while this species has not yet been documented in mainland Portugal, it has been recorded in Madeira and Azores archipelagos [116]. These results highlight the usefulness of metabarcoding techniques in dietary studies of insectivorous species, such as lizards, as tools for the detection of unrecorded arthropod species (e.g. Martins et al, 2022 [68]). 

However, the success of metabarcoding to complement biodiversity surveys is still hindered by the limited geographic and taxonomic coverage of the available DNA data in public repositories [117]. Moreover, reference DNA databases are subject to errors in accurate species identification or species misidentifications [118], that can become untraceable since there are no vouchers associated with deposited DNA sequences [119].

Although this was not the primary aim of our study, we identified other species not previously recorded in Portugal (*Acanthogethes fuscus, Diplazon tetragonus, Formica selysi, Monophadnoides ruficruris* and *Psilopa marginella)*. However, validating new localities for invertebrates is complex, since species distribution databases also contain taxonomic errors and geographical uncertainties, which can lead to overestimation or underestimation of species richness in certain areas [120]. Local databases are invaluable in this context, as they can provide more accurate and comprehensive information [121]. For example, while many species were not identified using the Global Biodiversity Information Facility (GBIF, http://www.gbif.org/), specialist sites for Portugal such as Naturdata (https://naturdata.com/) and Lusoborboletas (https://www.lusoborboletaspt.com/), allowed us to greatly refine the number of potentially new records for Portugal. Nonetheless, these potential new species records for Portugal should be treated with caution until they can be confirmed with actual specimens.

## Conclusion

This study allowed us to shed new light on the effects of wildfires on the diet of several populations of the generalist lizard, *Podarcis lusitanicus,* in a region regularly affected by these events, such as Northern Portugal. This study suggests that wildfires do not seem to affect the diet richness of *P. lusitanicus* but instead influence the composition of consumed prey items. Specifically, differences were observed between recently burned and unburned habitats, while lizards from areas burned 8 years ago and unburned did not show differences in arthropod prey composition. Our results also revealed that metabarcoding efforts to characterize diet of generalist species can provide important insights and help characterize local biodiversity.

## Supporting information

S1 Table *Podarcis lusitanicus* individuals examined at each site, with the corresponding fire regime-UN (unburned), BU16 (burned in 2016), and BU22 (burned in 2022)-along with sex (Female (F); Male (M)) and the respective weight (g) and snout-vent length (SVL, mm). (XLSX)

S2 Table Non-dietary data obtain from *Podarcis lusitanicus* diet samples. (XLSX)

S3 Table GLMM results for Fire regime site on NVDI values across Fire regime site, Sex and SVL (logSVL) on OTU and Family richness in *Podarcis lusitanicus.* (XLSX)

S4 Table ANOVA results for the effect of Fire regimesite (Locality as a random factor) and Sex on logSVL and logweight. Significant p-values (p < 0.05) are highlighted in bold.
(XLSX)

S5 Table Pairwise comparisons of *Podarcis* *lusitanicus* weight across fire regime sites. Significant p-values (p < 0.05) are highlighted in bold.
(XLSX)

S6 Table Dietary data from *Podarcis lusitanicus.* (XLSX)

S7 Table Frequency of ocurrence of OTUs across Fire regime sites from the diet of *Podarcis lusitanicus.* (XLSX)

S8 Table GLMM results for Fire regime site, Sex and SVL (logSVL) on OTU and Family richness in *Podarcis lusitanicus.* (XLSX)

S9 Table Permutation tests assessing the effects of predictor variables (Sex and Type site) on prey composition at the family level and evaluating the significance of RDA axes, with Site as a conditional factor. Significant p-values (p < 0.05) are highlighted in bold.
(XLSX)

S10 Table Frequency of occurrence of each OTU prey items present in the diet of *Podarcis lusitanicus* from areas burned in 2016 (BU16), burned in 2022 (BU22) and unburned (UN). (XLSX)

S11 Table Frequency of occurrence of families of prey items present in the diet of *Podarcis lusitanicus* from areas burned in 2016 (BU16), burned in 2022 (BU22) and unburned areas (UN). (XLSX)

S1 Fig Mean NDVI values across sites with different fire regimes. (XLSX)

S2 Fig Boxplot showing the differences between sexes regarding the logSVL (A) and log Weight (B) across the different types of fire regimes – areas burned in 2016 (BU16), burned in 2022 (BU22) and unburned areas (UN). (XLSX)

S3 Fig Linear regression of log-transformed SVL (logSVL) against log-transformed Weight (logWeight) for female (F) and male (M) individuals. (XLSX)

S4 Fig Histogram representing the frequency of occurrence of the arthropod orders (total of 26) consumed. (XLSX)

S5 Fig Estimated model coefficients and 95% confidence intervals (CIs) of the predictors used to model OTU and Family richness. Predictor variables had a significant effect when 95% confidence interval did not overlap 0. Areas burned in 2022 (BU22), and unburned areas (UNB) are compared with areas burned in 2016 regarding the fire regime sites.
(XLSX)

S6 Fig Rank abundance plot for the 10 more abundant OTUs found in *P. lusitanicus* diet, among the different fire regime sites – areas burned in 2016 (BU16), burned in 2022 (BU22) and unburned areas (UN). For a detailed list of the top 10 OTUs, refer to S10 Table.
(XLSX)

## References

[1] Turner MG. Disturbance and landscape dynamics in a changing world. Ecology. 2010;91(10):2833–49. doi: 10.1890/10-0097.1 21058545

[2] Chuvieco E, Giglio L, Justice C. Global characterization of fire activity: toward defining fire regimes from Earth observation data. Global Change Biology. 2008;14(7):1488–502. doi: 10.1111/j.1365-2486.2008.01585.x

[3] Catry FX, Rego FC, Bação FL, Moreira F. Modeling and mapping wildfire ignition risk in Portugal. Int J Wildland Fire. 2009;18(8):921. doi: 10.1071/wf07123

[4] Hosseini M, Keizer JJ, Pelayo OG, Prats SA, Ritsema C, Geissen V. Effect of fire frequency on runoff, soil erosion, and loss of organic matter at the micro-plot scale in north-central Portugal. Geoderma. 2016;269:126–37. doi: 10.1016/j.geoderma.2016.02.004

[5] Keeley JE, Bond WJ, Bradstock RA, Pausas JG, Rundel PW. Fire in Mediterranean ecosystems. Ecology, evolution and management. Cambridge University Press; 2011.

[6] Pausas JG, Verdú M. Plant persistence traits in fire‐prone ecosystems of the Mediterranean basin: a phylogenetic approach. Oikos. 2005;109(1):196–202. doi: 10.1111/j.0030-1299.2005.13596.x

[7] Syphard AD, Radeloff VC, Hawbaker TJ, Stewart SI. Conservation threats due to human-caused increases in fire frequency in Mediterranean-climate ecosystems. Conserv Biol. 2009;23(3):758–69. doi: 10.1111/j.1523-1739.2009.01223.x 22748094

[8] Izhaki I. The impact of fire on vertebrates in the Mediterranean Basin: an overview. Israel Journal of Ecology and Evolution. 2012;58(2–3):221–33.

[9] Sarà M, Bellia E, Milazzo A. Fire disturbance disrupts co‐occurrence patterns of terrestrial vertebrates in Mediterranean woodlands. Journal of Biogeography. 2006;33(5):843–52. doi: 10.1111/j.1365-2699.2006.01429.x

[10] Jolly CJ, Dickman CR, Doherty TS, van Eeden LM, Geary WL, Legge SM, et al. Animal mortality during fire. Glob Chang Biol. 2022;28(6):2053–65. doi: 10.1111/gcb.16044 34989061

[11] Santos X, Badiane A, Matos C. Contrasts in short- and long-term responses of Mediterranean reptile species to fire and habitat structure. Oecologia. 2016;180(1):205–16. doi: 10.1007/s00442-015-3453-9 26408003

[12] Santos X, Chergui B, Belliure J, Moreira F, Pausas JG. Reptile responses to fire across the western Mediterranean Basin. Conserv Biol. 2025;39(1):e14326. doi: 10.1111/cobi.14326 38949049 PMC11780213

[13] Moyo S. Community responses to fire: A global meta-analysis unravels the contrasting responses of fauna to fire. Earth. 2022;3(4):1087–111.

[14] Sutherland EF, Dickman CR. Mechanisms of recovery after fire by rodents in the Australian environment: a review. Wildlife Research. 1999;26(4):405–19.

[15] Nieman WA, van Wilgen BW, Radloff FG, Leslie AJ. A review of the responses of medium-to large-sized African mammals to fire. African Journal of Range & Forage Science. 2022;39(3):249–63.

[16] Hoare S. The possible role of predator-prey dynamics as an influence on early hominin use of burned landscapes. Evol Anthropol. 2019;28(6):295–302. doi: 10.1002/evan.21807 31652026

[17] Doherty TS, Geary WL, Jolly CJ, Macdonald KJ, Miritis V, Watchorn DJ, et al. Fire as a driver and mediator of predator-prey interactions. Biol Rev Camb Philos Soc. 2022;97(4):1539–58. doi: 10.1111/brv.12853 35320881 PMC9546118

[18] Alemany I, Pérez-Cembranos A, Pérez-Mellado V, Castro JA, Picornell A, Ramon C, et al. DNA metabarcoding the diet of *Podarcis* lizards endemic to the Balearic Islands. Curr Zool. 2022;69(5):514–26. doi: 10.1093/cz/zoac073 37637311 PMC10449427

[19] Mateus P, Fernandes PM. Forest fires in Portugal: dynamics, causes and policies. Forest context and policies in Portugal: present and future challenges. 2014. p. 97–115.

[20] San-Miguel-Ayanz J, Oom D, Artes T, Viegas DX, Fernandes P, Faivre N. Forest fires in Portugal in 2017. Science for disaster risk management. 2020;:413–30.

[21] Ferreira D, Pinho C, Brito JC, Santos X. Increase of genetic diversity indicates ecological opportunities in recurrent-fire landscapes for wall lizards. Sci Rep. 2019;9(1):5383. doi: 10.1038/s41598-019-41729-6 30926838 PMC6441018

[22] Meira Castro AC, Nunes A, Sousa A, Lourenço L. Mapping the Causes of Forest Fires in Portugal by Clustering Analysis. Geosciences. 2020;10(2):53. doi: 10.3390/geosciences10020053

[23] Pausas JG, Llovet J, Rodrigo A, Vallejo R. Are wildfires a disaster in the Mediterranean basin? - A review. Int J Wildland Fire. 2008;17(6):713. doi: 10.1071/wf07151

[24] Nunes AN. Regional variability and driving forces behind forest fires in Portugal an overview of the last three decades (1980–2009). Applied Geography. 2012;34:576–86. doi: 10.1016/j.apgeog.2012.03.002

[25] Gomes JFP. Forest fires in Portugal: how they happen and why they happen. International Journal of Environmental Studies. 2006;63(2):109–19. doi: 10.1080/00207230500435304

[26] Carretero MA. An integrated Assessment of a group with complex systematics: the Iberomaghrebian lizard genus *Podarcis* (Squamata, Lacertidae). Integr Zool. 2008;3(4):247–66. doi: 10.1111/j.1749-4877.2008.00102.x 21396075

[27] Harris DJ, Sá-Sousa P. Molecular phylogenetics of Iberian wall lizards (*Podarcis*): is *Podarcis hispanica* a species complex? Mol Phylogenet Evol. 2002;23(1):75–81. doi: 10.1006/mpev.2001.1079 12182404

[28] Carretero MA. From set menu toa la carte. Linking issues in trophic ecology of Mediterranean lacertids. Italian Journal of Zoology. 2004;71(sup2):121–33. doi: 10.1080/11250000409356621

[29] Speybroeck J, Beukema W, Bok B, Van Der Voort J. Field guide to the amphibians and reptiles of Britain and Europe. Bloomsbury publishing; 2016.

[30] Rato C, Sreelatha LB, Gómez‐Ramírez F, Carretero MA. A Pleistocene Biogeography in Miniature: The Small‐Scale Evolutionary History of *Podarcis lusitanicus* (Squamata, Lacertidae). Journal of Biogeography. 2024;52(1):186–98. doi: 10.1111/jbi.15026

[31] Geniez P, Sá-Sousa P, Guillaume CP, Cluchier A, Crochet P-A. Systematics of the *Podarcis hispanicus* complex (Sauria, Lacertidae) III: valid nomina of the western and central Iberian forms. Zootaxa. 2014;3794:1–51. doi: 10.11646/zootaxa.3794.1.1 24870311

[32] Ferreira D, Žagar A, Santos X. Uncovering the rules of (reptile) species coexistence in transition zones between bioregions. Salamandra. 2017;53(2):157–66.

[33] Diego-Rasilla FJ, Perez-Mellado V. Home range and habitat selection by *Podarcis hispanica* (Squamata, Lacertidae) in Western Spain. Folia Zoologica-Praha. 2003;52(1):87–98.

[34] Ferreira D, Mateus C, Santos X. Responses of reptiles to fire in transition zones are mediated by bioregion affinity of species. Biodivers Conserv. 2016;25(8):1543–57. doi: 10.1007/s10531-016-1137-3

[35] Calheiros T, Benali A, Pereira M, Silva J, Nunes J. Drivers of extreme burnt area in Portugal: fire weather and vegetation. Nat Hazards Earth Syst Sci. 2022;22(12):4019–37. doi: 10.5194/nhess-22-4019-2022

[36] Certini G, Moya D, Lucas-Borja ME, Mastrolonardo G. The impact of fire on soil-dwelling biota: A review. Forest Ecology and Management. 2021;488:118989. doi: 10.1016/j.foreco.2021.118989

[37] Ferrenberg S, Wickey P, Coop JD. Ground-dwelling arthropod community responses to recent and repeated wildfires in conifer forests of northern New Mexico, USA. Forests. 2019;10(8):667.

[38] Khayati ME, Chergui B, Taheri A, Fahd S, Santos X. Differential response to fire in ground vs. vegetation arthropod communities. J Insect Conserv. 2023;27(4):601–13. doi: 10.1007/s10841-023-00483-x

[39] Hope PR, Bohmann K, Gilbert MTP, Zepeda-Mendoza ML, Razgour O, Jones G. Second generation sequencing and morphological faecal analysis reveal unexpected foraging behaviour by *Myotis nattereri* (Chiroptera, Vespertilionidae) in winter. Frontiers in Zoology. 2014;11:1–15. 25093034 10.1186/1742-9994-11-39 PMC4108090

[40] Nielsen JM, Clare EL, Hayden B, Brett MT, Kratina P. Diet tracing in ecology: Method comparison and selection. Methods Ecol Evol. 2017;9(2):278–91. doi: 10.1111/2041-210x.12869

[41] da Silva LP, Mata VA, Lopes PB, Pereira P, Jarman SN, Lopes RJ, et al. Advancing the integration of multi-marker metabarcoding data in dietary analysis of trophic generalists. Mol Ecol Resour. 2019;19(6):1420–32. doi: 10.1111/1755-0998.13060 31332947 PMC6899665

[42] Pompanon F, Deagle BE, Symondson WOC, Brown DS, Jarman SN, Taberlet P. Who is eating what: diet assessment using next generation sequencing. Mol Ecol. 2012;21(8):1931–50. doi: 10.1111/j.1365-294X.2011.05403.x 22171763

[43] IUCN. The IUCN Red List of Threatened Species. Version 2025-1 Gland: International Union for Conservation of Nature; 2025. https://www.iucnredlist.org

[44] Berry S, Mackey B, Brown T. Potential applications of remotely sensed vegetation greenness to habitat analysis and the conservation of dispersive fauna. Pac Conserv Biol. 2007;13(2):120. doi: 10.1071/pc070120

[45] Chu T, Guo X. Remote Sensing Techniques in Monitoring Post-Fire Effects and Patterns of Forest Recovery in Boreal Forest Regions: A Review. Remote Sensing. 2013;6(1):470–520. doi: 10.3390/rs6010470

[46] Team RC. R: A language and environment for statistical computing. Vienna: R Foundation for Statistical Computing; 2013.

[47] Arnold EN. Resource partition among lacertid lizards in southern Europe. Journal of Zoology. 1987;1(4):739–82. doi: 10.1111/j.1096-3642.1987.tb00753.x

[48] Sagonas K, Pafilis P, Lymberakis P, Donihue CM, Herrel A, Valakos ED. Insularity affects head morphology, bite force and diet in a Mediterranean lizard. Biological Journal of the Linnean Society. 2014;112(3):469–84.

[49] Bas S. La comunidad herpetológica de Caurel: biogeografía y ecología. Amphibia-Reptilia. 1982;3(1):1–26.

[50] Pérez Mellado V. Estructura en una taxocenosis de Lacertidae (Sauria, Reptilia) del sistema central. Mediterránea Serie de Estudios Biológicos, N 6 (diciembre 1982); 1982. p 39–64.

[51] Kaliontzopoulou A, Adams DC, van der Meijden A, Perera A, Carretero MA. Relationships between head morphology, bite performance and ecology in two species of *Podarcis* wall lizards. Evolutionary Ecology. 2012;26:825–45.

[52] Braña F. Biogeografía, biología y estructura de nichos de la taxocenosis de saurios de Asturias. University of Ovideo; 1984.

[53] Galán P. Anfibios y reptiles del Parque Nacional de las Islas Atlánticas de Galicia: faunística, biología y conservación. Ministerio de Medo Ambiente, Secretaría General de Medio Ambiente, Organismo Autónomo Parques Nacionales; 2003.

[54] Kaliontzopoulou A, Carretero MA, Llorente GA. Morphology of the *Podarcis* wall lizards (Squamata: Lacertidae) from the Iberian Peninsula and North Africa: patterns of variation in a putative cryptic species complex. Zoological Journal of the Linnean Society. 2012;164(1):173–93.

[55] Karpestam E, Merilaita S, Forsman A. Reduced predation risk for melanistic pygmy grasshoppers in post-fire environments. Ecol Evol. 2012;2(9):2204–12. doi: 10.1002/ece3.338 23139879 PMC3488671

[56] Mola JM, Miller MR, O’Rourke SM, Williams NM. Wildfire reveals transient changes to individual traits and population responses of a native bumble bee *Bombus vosnesenskii*. J Anim Ecol. 2020;89(8):1799–810. doi: 10.1111/1365-2656.13244 32358976

[57] EL Khayati M, Chergui B, Barranco P, Fahd S, Ruiz JL, Taheri A, Santos X. Assessing the response of different soil arthropod communities to fire: A case study from northwestern Africa. Fire. 2023;6(5):206.

[58] Santos X, Mateos E, Bros V, Brotons L, De Mas E, Herraiz JA, et al. Is response to fire influenced by dietary specialization and mobility? A comparative study with multiple animal assemblages. PLoS One. 2014;9(2):e88224. doi: 10.1371/journal.pone.0088224 24516616 PMC3917858

[59] Barata M, Perera A, Harris DJ. Cryptic variation in the Moroccan high altitude lizard *Atlantolacerta andreanskyi* (Squamata: Lacertidae). African Journal of Herpetology. 2015;64(1):1–17. doi: 10.1080/21564574.2014.967815

[60] Perera A, Pérez-Mellado V, Carretero MA, Harris DJ. Variation between populations in the diet of the Mediterranean lizard *Lacerta perspicillata*. The Herpetological Journal. 2006;16(2):107–13.

[61] Sá-Sousa P, Vicente L, Crespo E. Morphological variability of *Podarcis hispanica* (Sauria: lacertidae) in Portugal. Amphibia-Reptilia. 2002;23(1):55–69.

[62] Maudet C, Luikart G, Dubray D, Von Hardenberg A, Taberlet P. Low genotyping error rates in wild ungulate faeces sampled in winter. Molecular Ecology Notes. 2004;4(4):772–5. doi: 10.1111/j.1471-8286.2004.00787.x

[63] Vamos EE, Elbrecht V, Leese F. Short COI markers for freshwater macroinvertebrate metabarcoding. Metabarcoding and Metagenomics. 2017;1:e14625.

[64] Elbrecht V, Braukmann TWA, Ivanova NV, Prosser SWJ, Hajibabaei M, Wright M, et al. Validation of COI metabarcoding primers for terrestrial arthropods. PeerJ. 2019;7:e7745. doi: 10.7717/peerj.7745 31608170 PMC6786254

[65] Tournayre O, Leuchtmann M, Filippi-Codaccioni O, Trillat M, Piry S, Pontier D, et al. In silico and empirical evaluation of twelve metabarcoding primer sets for insectivorous diet analyses. Ecol Evol. 2020;10(13):6310–32. doi: 10.1002/ece3.6362 32724515 PMC7381572

[66] Rato C, Dellinger T, Carretero MA. Dietary variation is driven by landscape heterogeneity in an insular omnivorous endemic lizard, revealed by DNA metabarcoding. Diversity. 2022;14(12):1078.

[67] Galão A, Soto EJ, Nunes J, Pedroso NM, Rocha R, Rato C. When pets go wild: Integrating DNA metabarcoding and morphological analyses to investigate the impacts of free-ranging cats (*Felis catus*) on oceanic islands. Biological Conservation. 2025;305:111089.

[68] Martins B, Silva-Rocha I, Mata VA, Gonçalves Y, Rocha R, Rato C. Trophic interactions of an invasive gecko in an endemic-rich oceanic island: insights using DNA metabarcoding. Frontiers in Ecology and Evolution. 2022;10:1044230.

[69] Zhang J, Kobert K, Flouri T, Stamatakis A. PEAR: a fast and accurate Illumina Paired-End reAd mergeR. Bioinformatics. 2014;30(5):614–20. doi: 10.1093/bioinformatics/btt593 24142950 PMC3933873

[70] Boyer F, Mercier C, Bonin A, Le Bras Y, Taberlet P, Coissac E. obitools: a unix-inspired software package for DNA metabarcoding. Mol Ecol Resour. 2016;16(1):176–82. doi: 10.1111/1755-0998.12428 25959493

[71] Rognes T, Flouri T, Nichols B, Quince C, Mahé F. VSEARCH: a versatile open source tool for metagenomics. PeerJ. 2016;4:e2584. doi: 10.7717/peerj.2584 27781170 PMC5075697

[72] Mata VA, Amorim F, Corley MFV, McCracken GF, Rebelo H, Beja P. Female dietary bias towards large migratory moths in the European free-tailed bat (*Tadarida teniotis*). Biol Lett. 2016;12(3):20150988. doi: 10.1098/rsbl.2015.0988 27009885 PMC4843218

[73] Evans HK, Bunch AJ, Schmitt JD, Hoogakker FJ, Carlson KB. High-throughput sequencing outperforms traditional morphological methods in Blue Catfish diet analysis and reveals novel insights into diet ecology. Ecol Evol. 2021;11(10):5584–97. doi: 10.1002/ece3.7460 34026031 PMC8131796

[74] Frøslev TG, Kjøller R, Bruun HH, Ejrnæs R, Brunbjerg AK, Pietroni C, et al. Algorithm for post-clustering curation of DNA amplicon data yields reliable biodiversity estimates. Nat Commun. 2017;8(1):1188. doi: 10.1038/s41467-017-01312-x 29084957 PMC5662604

[75] Buchner D, Leese F. BOLDigger – a Python package to identify and organise sequences with the Barcode of Life Data systems. MBMG. 2020;4. doi: 10.3897/mbmg.4.53535

[76] Kearse M, Moir R, Wilson A, Stones-Havas S, Cheung M, Sturrock S, et al. Geneious Basic: an integrated and extendable desktop software platform for the organization and analysis of sequence data. Bioinformatics. 2012;28(12):1647–9. doi: 10.1093/bioinformatics/bts199 22543367 PMC3371832

[77] Costa GC, Vitt LJ, Pianka ER, Mesquita DO, Colli GR. Optimal foraging constrains macroecological patterns: body size and dietary niche breadth in lizards. Global Ecology and Biogeography. 2008;17(5):670–7. doi: 10.1111/j.1466-8238.2008.00405.x

[78] R Core Team R. R: A language and environment for statistical computing. Vienna, Austria: R Foundation for Statistical Computing; 2013.

[79] Russell L. Emmeans: Estimated marginal means, aka least-squares means. R package version. 2018.

[80] Hsieh TC, Ma KH, Chao A. iNEXT: an R package for rarefaction and extrapolation of species diversity (Hill numbers). Methods Ecol Evol. 2016;7(12):1451–6. doi: 10.1111/2041-210x.12613

[81] MacGregor-Fors I, Payton ME. Contrasting diversity values: statistical inferences based on overlapping confidence intervals. PLoS One. 2013;8(2):e56794. doi: 10.1371/journal.pone.0056794 23437239 PMC3577692

[82] Chao A, Jost L. Coverage-based rarefaction and extrapolation: standardizing samples by completeness rather than size. Ecology. 2012;93(12):2533–47. doi: 10.1890/11-1952.1 23431585

[83] Wickham H. ggplot2: Elegant Graphics for Data Analysis. New York, NY, USA: Springer-Verlag; 2016.

[84] Oksanen J, Simpson G, Blanchet F, Kindt R, Legendre P, Minchin P. vegan: Community ecology package. 2.6–2 ed. CRAN; 2022.

[85] Martinez Arbizu P. pairwiseAdonis: Pairwise multilevel comparison using adonis. R package version 04; 2020. p. 1.

[86] Carretero MÁ, Galán P, Salvador Milla A. Lagartija lusitana–*Podarcis lusitanicus* Geniez, Sá-Sousa, Guillaume, Cluchier y Crochet, 2014: CSIC - Museo Nacional de Ciencias Naturales (MNCN); 2022.

[87] Ingerson-Mahar J. Relating diet and morphology in adult carabid beetles. In: H J, editor. The agroecology of carabid beetles. Andover, UK: Intercept. 2002. p. 111–36.

[88] Katsimanis N, Dretakis M, Akriotis T, Mylonas M. Breeding bird assemblages of eastern Mediterranean shrublands: composition, organisation and patterns of diversity. Journal of Ornithology. 2006;147:419–27.

[89] Moreira F, Delgado A, Ferreira S, Borralho R, Oliveira N, Inácio M, et al. Effects of prescribed fire on vegetation structure and breeding birds in young *Pinus pinaster* stands of northern Portugal. Forest Ecology and Management. 2003;184(1–3):225–37. doi: 10.1016/s0378-1127(03)00214-7

[90] Suárez-Seoane S, Osborne PE, Baudry J. Responses of birds of different biogeographic origins and habitat requirements to agricultural land abandonment in northern Spain. Biological Conservation. 2002;105(3):333–44.

[91] Viljur M-L, Abella SR, Adámek M, Alencar JBR, Barber NA, Beudert B, et al. The effect of natural disturbances on forest biodiversity: an ecological synthesis. Biol Rev Camb Philos Soc. 2022;97(5):1930–47. doi: 10.1111/brv.12876 35808863

[92] Robinson NM, Leonard SW, Ritchie EG, Bassett M, Chia EK, Buckingham S. Refuges for fauna in fire‐prone landscapes: their ecological function and importance. Journal of Applied Ecology. 2013;50(6):1321–9.

[93] Steinitz O, Shohami D, Ben-Shlomo R, Nathan R. Genetic consequences of fire to natural populations. Israel Journal of Ecology and Evolution. 2012;58(2–3):205–20.

[94] Banks SC, Dujardin M, McBurney L, Blair D, Barker M, Lindenmayer DB. Starting points for small mammal population recovery after wildfire: recolonisation or residual populations?. Oikos. 2010;120(1):26–37. doi: 10.1111/j.1600-0706.2010.18765.x

[95] Watson SJ, Taylor RS, Nimmo DG, Kelly LT, Clarke MF, Bennett AF. The influence of unburnt patches and distance from refuges on post‐fire bird communities. Animal Conservation. 2012;15(5):499–507. doi: 10.1111/j.1469-1795.2012.00542.x

[96] Kelly LT, Brotons L, Giljohann KM, McCarthy MA, Pausas JG, Smith AL. Bridging the Divide: Integrating Animal and Plant Paradigms to Secure the Future of Biodiversity in Fire-Prone Ecosystems. Fire. 2018;1(2):29. doi: 10.3390/fire1020029

[97] Whelan R, Rodgerson L, Dickman C, Sutherland E. Critical life cycles of plants and animals: developing a process-based understanding of population changes in fire-prone landscapes. In: Bradstock RA, Gill AM, editors. Flammable Australia: The fire regimes and biodiversity of a continent. Cambridge: Cambridge University Press. 2002. p. 94–124.

[98] Podgaiski LR, Joner F, Lavorel S, Moretti M, Ibanez S, Mendonça M de S Jr, et al. Spider trait assembly patterns and resilience under fire-induced vegetation change in South Brazilian grasslands. PLoS One. 2013;8(3):e60207. doi: 10.1371/journal.pone.0060207 23555927 PMC3610671

[99] Parmenter RR, Kreutzian M, Moore DI, Lightfoot DC. Short-term effects of a summer wildfire on a desert grassland arthropod community in New Mexico. Environ Entomol. 2011;40(5):1051–66. doi: 10.1603/EN11047 22251717

[100] Riechert SE, Reeder WG. Effects of fire on spider distribution in southwestern Wisconsin prairies. Proceedings of the Second Midwest Prairie Conference, Madison, Wisconsin, 1972.

[101] Meyer CK, Whiles MR, Charlton RE. Life History, Secondary Production, and Ecosystem Significance of Acridid Grasshoppers in Annually Burned and Unburned Tallgrass Prairie. American Entomologist. 2002;48(1):52–61. doi: 10.1093/ae/48.1.52

[102] Apigian KO, Dahlsten DL, Stephens SL. Fire and fire surrogate treatment effects on leaf litter arthropods in a western Sierra Nevada mixed-conifer forest. Forest Ecology and Management. 2006;221(1–3):110–22. doi: 10.1016/j.foreco.2005.09.009

[103] Moretti M, Obrist MK, Duelli P. Arthropod biodiversity after forest fires: winners and losers in the winter fire regime of the southern Alps. Ecography. 2004;27(2):173–86. doi: 10.1111/j.0906-7590.2004.03660.x

[104] Manuel Vidal-Cordero J, Arnan X, Rodrigo A, Cerdá X, Boulay R. Four-year study of arthropod taxonomic and functional responses to a forest wildfire: Epigeic ants and spiders are affected differently. Forest Ecology and Management. 2022;520:120379. doi: 10.1016/j.foreco.2022.120379

[105] Pressler Y, Moore JC, Cotrufo MF. Belowground community responses to fire: meta‐analysis reveals contrasting responses of soil microorganisms and mesofauna. Oikos. 2018;128(3):309–27. doi: 10.1111/oik.05738

[106] Cancelado R, Yonke TR. Effect of prairie burning on insect populations. Journal of the Kansas Entomological Society. 1970;:274–81.

[107] Nagel HG. Effect of spring prairie burning on herbivorous and non-herbivorous arthropod populations. Journal of the Kansas Entomological Society. 1973:485–96.

[108] Andersen AN. Responses of Ground-Foraging Ant Communities to Three Experimental Fire Regimes in a Savanna Forest of Tropical Australia. Biotropica. 1991;23(4):575. doi: 10.2307/2388395

[109] Vasconcelos HL, Maravalhas JB, Cornelissen T. Effects of fire disturbance on ant abundance and diversity: a global meta-analysis. Biodivers Conserv. 2016;26(1):177–88. doi: 10.1007/s10531-016-1234-3

[110] Umaña-Castro R, Cambronero-Granados JA, Carvajal-Sánchez JP, Alfaro-Montoya J. Identificación molecular y distribución potencial del anfípodo terrestre *Talitroides topitotum* (Crustacea: Amphipoda: Talitridae) en Costa Rica. Acta Biológica Colombiana. 2018;23(1):104–15.

[111] Alfaro-Montoya J, Umaña Castro R. Primer registro e histología básica del anfípodo terrestre *Talitroides topitotum* (Amphipoda: Talitridae), introducido en las zonas montañosas de Heredia, Costa Rica. Archivos. 2013;5.

[112] Brisotto G, Ayres-Peres L, Santos S. Estrutura populacional de *Talitroides topitotum* (Crustacea: Amphipoda: Talitridae) em Santa Maria, região central do Rio Grande do Sul, Brasil. Iheringia Série Zoologia. 2022;112:e2022017.

[113] Daneliya ME, Wowor D. Cosmopolitan landhopper *Talitroides topitotum* (Crustacea, Amphipoda, Talitridae) in Java, Indonesia. cl. 2016;12(4):1933. doi: 10.15560/12.4.1933

[114] Lopes OL, Masunari S. Biologia reprodutiva de *Talitroides topitotum* (Burt) (Crustacea, Amphipoda, Talitridae) na Serra do Mar, Guaratuba, Paraná, Brasil. Rev Bras Zool. 2004;21(4):755–9. doi: 10.1590/s0101-81752004000400004

[115] Fernandes P, Antunes C, Pinho P, Máguas C, Correia O. Natural regeneration of *Pinus pinaster* and *Eucalyptus globulus* from plantation into adjacent natural habitats. Forest Ecology and Management. 2016;378:91–102. doi: 10.1016/j.foreco.2016.07.027

[116] Secretariat G. GBIF Backbone Taxonomy Checklist dataset; 2022. Flammulina ononidis. 2024.

[117] de Sousa LL, Silva SM, Xavier R. DNA metabarcoding in diet studies: Unveiling ecological aspects in aquatic and terrestrial ecosystems. Environmental DNA. 2019;1(3):199–214. doi: 10.1002/edn3.27

[118] Harris DJ. Can you bank on GenBank? Trends in Ecology & Evolution. 2003;18(7):317–9. doi: 10.1016/s0169-5347(03)00150-2

[119] Santos AM, Branco M. The quality of name-based species records in databases. Trends Ecol Evol. 2012;27(1):6–7. doi: 10.1016/j.tree.2011.10.004 22056882

[120] Maldonado C, Molina CI, Zizka A, Persson C, Taylor CM, Albán J, et al. Estimating species diversity and distribution in the era of Big Data: to what extent can we trust public databases?. Glob Ecol Biogeogr. 2015;24(8):973–84. doi: 10.1111/geb.12326 27656106 PMC5012125

[121] Meier R, Dikow T. Significance of Specimen Databases from Taxonomic Revisions for Estimating and Mapping the Global Species Diversity of Invertebrates and Repatriating Reliable Specimen Data. Conservation Biology. 2004;18(2):478–88. doi: 10.1111/j.1523-1739.2004.00233.x

